# HIV prevalence and TB in migrant miners communities of origin in Gaza Province, Mozambique: The need for increasing awareness and knowledge

**DOI:** 10.1371/journal.pone.0231303

**Published:** 2020-04-08

**Authors:** Cynthia Semá Baltazar, Yara Voss DeLima, Helena Ricardo, Carlos Botão, Denise Chitsondzo Langa, Paulino da Costa, Diosdélio Malamule, Ângelo Augusto, Sofia Viegas, Nkechi Obisie-Nmehielle, Laura Tomm-Bonde, Francis Bwambale Mulekya

**Affiliations:** 1 Instituto Nacional de Saúde, Maputo, Mozambique; 2 Centro de Investigação em Saúde da Polana Caniço (CISPOC), Instituto Nacional de Saúde, Maputo, Mozambique; 3 International Organization for Migration, Pretoria, South Africa; 4 International Organization for Migration, Maputo, Mozambique; Agencia de Salut Publica de Barcelona, SPAIN

## Abstract

**Background:**

As part of ongoing efforts to generate evidence needed on HIV and tuberculosis (TB) to inform policies and programs aimed to improve the health outcomes of migrants and communities affected by migration and mining, a preliminary investigation was conducted through a biological and behavioral (BBS) approach related to HIV and TB in two communities of origin of migrant mineworkers in Gaza Province. The main objective was to determine the prevalence of HIV and the rates of asymptomatic infection by TB, and the social and behavioral risk factors associated.

**Methods:**

A cross-sectional survey was conducted from May to June 2017 using a simple random sampling methodology. Eligible participants were individuals who were living in the community at the time the survey was conducted, which included adult mine workers and members of their families aged 18 and above. A socio-behavioral questionnaire was administered, blood specimens were collected for HIV testing (Determine/Unigold) and sputum for TB (GeneXpert MTB/RIF) was collected. The statistical analysis was performed using the R studio software to produce means, proportion and odds ratio at 95% confidence intervals.

**Results:**

A total of 1012 participants were enrolled, 75.2% were females, with a median age of 34. The overall prevalence of HIV found in the two communities was 24.2% (CI: 21.6–27.0) and was higher in the rural community (31.6%; 95% CI: 27.0–35.3). The prevalence of active TB was found to be 0.3% (n = 3) while 7.5% of the participants self-reported to have been previously diagnosed with TB at some point in their life. Only 2.8% of participants had knowledge of the basic principles of TB transmission. Condom use at last sexual intercourse with a regular partner was low among both sexes (17.3% male and 12.6% female). A considerable proportion of participants had not been aware of their HIV positive serostatus(31.1% female and 25.0% male). About 1/3 of the participants had had a history of STIs.

**Conclusion:**

The results of this survey confirm a high prevalence of HIV in communities of origin of migrant miners in Gaza province. Findings also demonstrated low levels of awareness/ knowledge and prevention of TB and HIV. It is important to strengthen strategies that encourage regular HIV testing and TB screening. Appropriate communication interventions on methods of transmission and prevention of HIV and TB in these communities must be intensified, as well as ensuring ongoing linkage to TB and HIV social and healthcare services.

## Introduction

The South African mining industry has been a major contributor to the economy of the surrounding countries of the Southern African Development Community (SADC) due to job creation and tariffs paid to the countries sending their workforce to South Africa [1,2]. Indeed, mining companies have contributed towards improved social development. [3] Migration patterns related to working away from one’s home country have contributed to HIV transmission in the region. found to be significantly linked to the spread of HIV/AIDS at a population level in Africa, especially in Sub-Saharan region [4].

Migrant miners largely represent a group of sexually active men who are separated from their regular partners for extended periods. Research has noted that the migrant nature of work is characterized by access to commercial sex work, high alcohol use and low condom use [5]. This may contribute to the increased vulnerability to female partners residing in their communities of origin. It is widely recognized that the prevalence and the vulnerability to HIV infection is more pronounced among the miners, their families and communities than in the general population [2,3,6–9]. There are several environmental and individual structural factors contributing to the increase of vulnerability to HIV. Of those, the structural factors, such as the sectoral policies, national and regional labour, migration patterns and health access, have a global impact on the epidemic of HIV, mainly because of the conditions in which the mining sector operates [10].

Mozambique has one of the highest HIV prevalence in the world [11], 13.2% among adults aged 15–49 years of age [12]. The most significant risk factors for HIV high prevalence in the country are associated with multiple partnerships and low rates of condom use among heterosexual partners. Additional factors that drive the epidemic include a high rate of vertical transmission, high population mobility, and behaviors such as early sexual initiation, multiple partners and a low rate of condom use [12–14].

Tuberculosis is an important co-morbidity associated with HIV in the Sub-Saharan region. Since their beginning, more than a 100 years ago, the gold and platinum underground mines in South Africa have had some of the highest TB incidence rates in the world [7,15]. The working conditions inside mines have been found to be a high-risk environment for TB transmission due to the exposure of silica and dust, leading to silicosis which leads to having a higher risk of developing tuberculosis. The general living conditions of the miners combined with migration also facilitates the transmission of TB to the general community with serious implications for prevention and control strategies [1].

Mozambican miners working in South Africa were recognized by the government of Mozambique as a high-risk group in the National Strategic Plan for HIV and AIDS Response (PEN III) (Council of Ministers, 2010). They are currently considered an important target group for HIV prevention and treatment programs [6] and by the National TB Control Programmed.

Biological and Behavioral Surveys (BSS) provide important data about the prevalence of HIV and other diseases as well as the behavioral, social and environmental issues that affect disease transmission in a specific population. In addition, they also provide an opportunity to detected undiagnosed TB and HIV cases in the community. Estimating HIV and TB prevalence and identifying the risk factors associated with disease transmission in the communities of origin of migrant mineworkers will contribute to a better understand of the health needs and behaviors of Mozambican workers in mines, as well as the service needs of members of their communities of origin. Such data is useful to guide and inform policy, design interventions for HIV/AIDS and TB control, to correctly targeted and allocate the appropriate resources for Mozambican miners in South Africa.

## Methods

### Study setting and design

A descriptive cross-sectional survey was conducted from 29 May to 23 June 2017 in two neighbourhoods within the Xai-Xai district in Gaza Province, Mozambique. Gaza is the province which currently supplies the majority of migrant mineworkers to work in South Africa [6]. Gaza also has the highest HIV prevalence among the general population [12,6].

A formative assessment was conducted 4 months prior to the implementation of the survey and during which the communities of Muzingane and Patrice Lumumba were identified as survey sites given that more than 60% of households had active or ex-mineworkers household members. (Reference: TEBA and the Mozambican miner’s association—AMIMO). Patrice Lumumba represents an urban setting and Muzigane a rural setting.

The sample size was calculated assuming an HIV prevalence of 24% with a significance level of 95% and power of 80%, with a no-response of 10%. The result was approximately 1,000 respondents, which corresponded to approximately 250–300 households.

Data were collected at household level in both communities. In the two neighbourhoods houses were selected systematically using a random sampling methodology with a starting point and sampling interval of n  =  3. We identified the first house from a defined central point and every 3^rd^ house moving in one cardinal direction was therefore selected for participation. If there was no response at the selected house or if they refused participation, then the next house was selected until sample size was reached. We worked with community leaders beforehand to ensure successful community mobilization and engagement in the study. There were no direct incentives given to participate on the survey besides the indirect incentive of providing free testing and counselling at household level. All data and samples were collected in the participating household.

### Participant eligibility

Individuals were eligible to participate if they were 18 years of age or older, living in the communities of Muzingane and Patrice Lumumba, in Gaza Province and able to provide informed consent for the study.

### Behavioral questionnaire

Behavioral data was collected through a standardized questionnaire that included questions aligned with national and global HIV and TB indicators. The questionnaire collected data on topics that included demographic and behavioural information related to HIV, TB, STIs and access and demand for health services.

Interviewers were trained to administer the questionnaire verbally in local language (Changana), if necessary. The questionnaire was programmed electronically in ODK (Open Data Kit, version 2.0) and administered by interviewers using a tablet (OpenHDS mobile), stored in ODK-Collect and synchronized with the central database through the ODK-Aggregate server.

### Sample collection and testing

Participants were offered on-site HIV testing to know their serostatus. All HIV testing procedures followed the National guidelines on HIV testing services algorithm [16]. The guidelines use an algorithm that initially screens with Determine^™^ HIV-1/2 (Alere Medical, Japan), and confirmation is done with Uni-Gold HIV^™^ (Trinity Biotech, Ireland). All participants participated in counselling sessions before and after the testing on a voluntary basis [20]. Participants with seropositive results or repeatedly indeterminate (considered negative for HIV prevalence estimates in this study) results were referred to the nearest health facility for linkage to care and treatment services for HIV.

For the diagnosis of TB, one sputum sample was collected on-site after the questionnaire was administered. The sample was requested from all study participants, with or without TB symptoms, except for participants who reported being on TB treatment. Procedures for collecting sputum samples, packaging and shipping followed the standard operational procedures in place from the National TB Reference Laboratory. The cold chain was maintained using frozen ice-packs in cooler boxes during sample transportation. At the reference laboratory in Maputo testing was performed using GeneXpert^®^ MTB/RIF.

### Data analysis

The data was cleaned, and the recoded database was exported to conduct descriptive and analytical statistics analysis with R studio software (Version 7.1.38 *R Foundation for Statistical Computing*). We calculated the proportions of socio-demographic, behavioral variables, and also the prevalence of HIV. Significance was assesses using Chi-square test. The associations between the prevalence of HIV and risk factors were assessed by bivariate regression analysis. Odds ratios (ORs) and 95% confidence intervals (CIs) were calculated for each association. Significant variables were retained in the multivariate logistic regression. The P-value ≤ 0.05 was considered statistically significant.

### Ethical considerations

Fieldworkers asked to speak to the head of each household first, before presenting the survey and approaching each household member. Each participant underwent a process of informed consent and were requested to provide written consent to allow the questionnaire to be administered and only tested for HIV and TB separately if consented. Face-to-face interviews were conducted privately with consenting participants in the household in an area selected by the participant, respecting their privacy and confidentiality. To protect the identity of participants, no personal identifier information was collected into the database. All data and biological samples were identified during collection, transportation and storage through individual participant codes (CIP) and an attributed serial individual laboratory number.

The research protocol was approved by the Institutional Committee of Bioethics for Health (CIBS) at the National Institute of Health (INS) and by the National Committee for Bioethics for Health (CNBS) of Mozambique (Ref. 105/CNBS/17). The data collection team benefited from protocol and SOP (standard operating procedure) training and signed a confidentiality agreement before starting their duties during the investigation.

## Results

Of the 1026 eligible household members, 1013 (98.7%) agreed to participate and complete the questionnaire, as well as undergo HIV and TB testing. One questionnaire was removed during the data cleaning, having thus obtained a sample size of 1012.

### General socio-demographic characteristics

The median age of the interviewed population was 34 years and most participants were between 25–39 years (37.1%). The majority of participants were female (75.2%) and were living in the community for more than 3 years (97.3%). Nearly half of the participants (49.2%) completed at primary level education and 10.2% had never attended school. Of the total number of participants interviewed, 5.8% reported having worked in a South Africa mine, and of these a little more than half (55.8%) worked for more than 10 years. Twenty-seven percent currently lived in the same house with a miner or an ex-miner, of which 54.5% were spouses of miners or ex-miners (Table 1).

**Table 1:** Socio-demographic characteristics of participants, in miners community of origin, Gaza Province, Southern of Mozambique, 2017.

Characteristic	N = 1012	%	CI 95%
**Age Range**			
18–24	295	29.2	26.4–32.1
25–39	375	37.1	34.1–40.1
40–49	150	14.8	12.7–17.2
≥ 50	192	18.9	16.6–21.6
*Median [minimum*, *maximum]*	34 (18, 64)	-	-
**Gender**			
Female (vs male)	761	75.2	72.4–77.8
**Nationality**			
Mozambican	1011	99.9	99.3–100
**Number of years lived in Gaza Province**			
Less than 1 year	5	0.5	0.2–1.2
1 to 3 years	22	2.2	1.4–3.3
More than 3 years	984	97.3	96.1–98.2
**Level of education**			
Never been to school	103	10.2	8.4–12.3
Primary or literacy	495	49.2	46.0–52.3
Secondary Education	382	37.9	34.9–41.0
Higher education or professional training	27	2.7	1.8–3.9
**Religion**			
Protestant	419	41.5	38.4–44.5
Catholic	152	15.0	12.9–17.4
Muslim	5	0.5	0.1–1.2
None	102	10.1	8.3–12.1
Other	333	32.9	30.0–35.8
**The main source of income for the family**			
Formal employment	474	47.0	43.8–50.1
Contributions from relatives	205	20.3	17.9–22.9
No income	26	2.6	1.7–3.8
Other sources ^a^	304	30.0	27.3–34.6
*Missing*	*3*		
**Worked in a mine in South Africa**			
Yes	59	5.8	4.5–7.5
**Total number of years worked in the mines in South Africa (n = 59)** ^b^			
Less than 10 years	23	44.2	28.4–55.0
More than 10 years	29	55.8	38.2–65.2
*Missing*	*7*		
**Currently lives in the same house with a miner or ex-miner who works/worked in South Africa mines**			
Yes	278	27.5	30.7–58.6
**Relation with a miner or ex-miner who currently lives in the same house (n = 278)** ^c^			
Spouse	151	54.5	48.4–60.5
Partner (sexual /boyfriend/girlfriend)	2	-	-
Other relative	124	44.8	38.8–50.8
*Missing*	*1*		

^a^ Other sources includes:income from informal street trading, gardening, cross-border trade and craftsmanship

^b^ Only for those who have already worked in South Africa

^c^ Only for those who currently live in the same house with a miner or ex-miner

### Marital status, sexual history and risk behaviour

The distribution of both female and male respondents according to their marital status, sexual history and risk behaviour is shown in Table 2. More than half of both female and male participants (60.5% and 51.0%, respectively) were married or living in a union. Forty-five percent of male reported having at least two sexual partners in the last 12 months. Seven out of ten (72.4%) female participants reported that their spouse was the main sexual partner, while for male participants this was 51.2%. Sexual debut before the age of 18 was reported by the overwhelming majority of participants (82.9%). Little more than half of the male participants (53.6%) reported having had sex under the influence of alcohol in the last 12 months. Around one third of both female and male participants (33.0% and 35.7%, respectively) reported that they had an STI diagnosis or symptoms in the last 12 months preceding the survey.

**Table 2:** Marital status and sexual risk behaviours among sexually active participants in miners community of origin, Gaza Province, Southern of Mozambique, 2017(N = 1012).

Characteristic	Female (n = 761)			Male (n = 251)		
	N	%	CI 95%	N	%	CI 95%
**Marital status**						
Single/never married	126	16.5	14.0–19.4	94	37.5	31.5–43.8
Married/living in a civil union	460	60.5	56.9–63.9	128	51.0	44.6–57.3
Widowed/divorced/separated	175	23.0	20.1–26.2	29	11.5	8.0–16.3
**Primary sexual partner in the last month (n = 598 vs n = 218)**^a^						
Boyfriend/Girlfriend	162	27.1	23.6–30.9	111	46.1	39.4–53.0
Partner/spouse	433	72.4	58.6–75.9	100	51.2	44.3–58.0
Transactional partner	2	-	-	6	2.8	1.1–6.2
Casual acquaintance (s) or casual partner (s)	1	-	-	1	-	-
**Number of sexual partners in the last 12 months (n = 599 vs n = 222)**^b^						
1	563	94.1	63.4–70.0	119	54.6	47.7–61.3
2	35	5.9	4.2–8.1	99	45.5	38.7–52.3
±3	1	-	-	4	-	-
**Age of first sexual intercourse**						
Less than 18 years	581	82.9	79.8–85.6	23	10.6	7.0–15.7
18 years or more	120	17.1	14.4–20.2	139	64.1	57.2–70.4
*Missing*	*60*			*89*		
**Forced to have sex in the past 12 months/ Unwanted sexual experience (coercive) in the past 12 months**						
Yes (vs no)	41	5.4	5.0–9.2	na	na	na
**Sex under the influence of alcohol in the past 12 months**						
Yes (vs no)	51	28.6	22.2–36.0	75	53.6	24.4–36.0
**Age difference of most recent sexual partner(n = 599 vs n = 222)**^b^						
10± years older	379	65.4	61.2–69.2	142	64.0	57.2–70.2
10± years younger	37	6.4	4.6–8.8	69	31.1	25.1–37.7
Not 10 or more years older or younger	164	27.4	24.7–32.2	10	4.5	2.3–8.4
*Missing*	*19*			*1*		
**Number of sexual partners in the last month (n = 598 vs n = 218)**^a^						
1	563	94.1	91.9–95.8	119	54.6	47.7–61.3
≥2	35	5.9	4.2–8.1	99	45.6	38.7–51.3
**Self-reported STIdiagnosis or symptoms in the past 12 months**						
Yes (vs no)	232	33.0	30.0–36.6	84	35.7	29.7–42.3
*Missing*	*1*			*1*		

^a^ Only for those who have sexual partner in the last month

^b^ Only for those who have sexual partner in the last year

na—not applicable

### HIV-related knowledge, perceptions and attitudes

The majority of participants (76.1% female and 79.7% male) stated that a person can protect themselves from HIV by using the condom correctly and consistently. Nearly half (58.9% of female and 68.9% male) stated that person can be protected by having only one uninfected sexual partner. However, 36.7% of female and 34.8% of male participants stated that HIV can be transmitted by a mosquito bite. The majority (68.0% female and 73.3% of male) stated that a person cannot get HIV from receiving injections with a needle that has already been used by another person, and nine out of ten participants (89.1% female and 90.4% of male) affirmed that a person can contract HIV if they share a meal with someone who is infected. Thirty one percent of female participants and 25.0% of male participants who tested positive for HIV in this survey were not aware of their HIV status (Table 3).

**Table 3:** Knowledge, attitudes and perceptions about HIV in miners community of origin, Gaza Province, Southern of Mozambique, 2017.

Characteristic	Female (n = 761)			Male (n = 251)		
	N	%	CI 95%	N	%	CI 95%
**A person can protect himself from HIV by correctly and consistently using a condom**						
Yes (vs no)	579	76.1	72.9–79.0	200	79.7	74.1–84.4
Don't know	69	9.1	7.2–11.4	14	5.6	3.2–9.4
**A person can protect himself from HIV by having only one uninfected sexual partner**						
Yes (vs no)	448	58.9	55.3–62.4	173	68.9	62.7–74.5
Don't know	42	5.5	4.1–7.5	9	3.6	1.8–6.9
**A person can protect himself from HIV by sexual abstinence**						
Yes (vs no)	364	47.8	44.2–51.4	124	49.4	43.1–55.7
Don't know	42	5.5	4.1–7.5	10	4.0	2.0–7.4
**A person can get HIV by getting injections with a needle that has already been used by someone else**						
Yes (vs no)	146	19.2	16.5–22.2	41	16.3	12.1–21.6
Don't know	97	12.8	10.5–15.4	26	10.4	7.0–15.0
**HIV can be transmitted by a mosquito bite**						
Yes (vs no)	279	36.7	33.2–40.2	87	34.8	29.0–41.1
Don't know	151	19.8	17.1–22.9	30	12.0	8.4–16.8
**HIV can be transmitted by sharing of a meal with an infected person**						
Yes (vs no)	737	96.8	95.3–98.0	247	98.4	95.7–99.5
Don't know	16	2.1	1.2–3.5	3	1.2	0.3–3.7
**Should HIV positive student be in school**						
Yes (vs no)	722	94.9	93.0–96.3	241	96.0	92.6–98.0
Don't know	7	0.9	0.4–2.0	2	0.8	0.1–3.1
**If a family member becomes ill with HIV/AIDS would be willing to take care of him/her**						
Yes (vs no)	744	97.8	96.4–98.7	242	96.4	93.1–98.2
Don't know	4	0.5	0.2–1.4	2	0.8	0.1–3.1
**Should HIV positive teacher continue teaching**						
Yes (vs no)	711	93.5	91.4–95.0	236	94.4	90.6–96.8
Don't know	15	2.0	1.1–3.3	2	0.8	0.1–3.2
**Buying of food from an infected food-seller**						
Yes (vs no)	627	82.4	79.5–85.0	205	81.7	76.2–86.1
Don't know	11	1.4	0.8–2.7	7	2.8	1.2–5.9
**Would infection of a family member be kept a secret**						
Yes (vs no)	462	60.7	57.1–64.2	126	50.2	43.9–56.5
Don't know	9	1.2	0.6–2.3	6	2.4	1.0–5.4
**Ever tested for HIV**						
Yes (vs no)	690	90.9	88.6–92.8	185	74.3	68.3–79.5
*Missing*	*2*			*2*		
**Tested for HIV in the last 12 months**						
Yes (vs no)	326	61.3	57.0–65.4	82	59.9	51.1–68.0
*Missing*	*158*			*48*		
**Awareness of own HIV-positive status (n = 326 vs n = 82)**						
Was not aware of HIV-positive status (vs was aware) ^a^	49	31.1	24.0–38.9	6	25.0	10.6–47.1

^a^ Only for those who had a HIV test in the last 12 months

### HIV prevalence and risk factors

The overall HIV prevalence among participants was 24.2% (95% CI: 21.6–27.0). The prevalence was 31.6% (95% CI: 27.0–35.3) at Muzingane community (rural area) and 17.6% (95% CI: 14.4–21.1) in Patrice Lumumba neighborhood (urban area). The prevalence of HIV was higher in participants aged 40–49 years (32.7%; 95%CI: 24.2–39.2, p<0.01) and tended to decline with increasing levels of education of participants. HIV prevalence does not differ in those who currently live with a miner or ever lived with a miner (Table 4). In multivariate analysis, HIV was associated with living in the Muzigane neighborhood (aOR 1.6; 95%CI: 1.3–1.9), 25–39 age range (aOR 2.2; 95% CI: 1.3–3.6) 40–49 age range (aOR 2.2; 95%CI: 1.2–3.8) and primary level of education (aOR 1.5; 95%CI: 0.9–2.5).

**Table 4:** HIV Prevalence by socio-demographic characteristics and behavior characteristics in miners community of origin, Gaza Province, Southern of Mozambique, 2017.

	n/N	Prevalence	CI 95%	OR	CI 95%	p-value	aOR n =	95% CI	p-value
**Community**									
Muzingane	155/500	31.0	27.0–35.3	1.4	1.3–1.6	<0.01	1.6	1.3–1.9	<0.01
Patrice Lumumba	90/512	17.6	14.4–21.1			REF			
**Age group (ref: 18–24)**									
25–39	115/347	33.1	28.3–38.4	3.7	2.5–5.6	<0.01	2.2	1.3–3.6	<0.01
40–49	49/157	31.2	24.2–39.2	3.4	2.11–5.5	<0.01	2.2	1.2–3.8	<0.01
≥50	42/177	23.7	17.8–30.8	2.3	1.4–3.8	<0.01	1.6	0.8–2.9	0.15
**Female gender (ref: male)**	205/761	26.9	23.8–30.3	0.5	0.3–0.7	<0.01	1.6	0.9–2.6	0.09
**Level of education (ref: never attended school)**									
Primary or literacy	52/383	13.6	10.4–35.2	2.9	0.1–0.2	<0.01	1.5	0.9–2.5	0.01
Secondary Education	46/419	7.4	1.3–25.8	0.2	2.1–4.2	0.25			
**Religion**									
Catholic	25/152	14.4	11.1–23.5	1.0	0.6–1.7	0.97	-	-	-
Muslim	22/102	21.6	14.3–31.0	1.7	0.0–1.0	0.97	-	-	-
Other	99/331	29.9	25–35.2	1.5	0.9–2.6	1.60	-	-	-
None	99/419	23.6	19.7–28.0			REF			
**Worked in a south African mine**	16/59	27.1	16.7–40.5	1.2	0.6–2.1	0.59	-	-	-
**Self-reported STI diagnosis or symptoms in the past 12 months**	87/316	27.5	22.1–34.1	1.2	0.9–1.7	0.12	-	-	-
**Ever tested for HIV, lifetime**	223/875	25.5	22.6–28.5	1.9	1.2–3.3	0.01	1.4	0.7–2.7	0.28
**Currently lives with a miner or former miner**	121/538	22.5	19.1–26.3	1.2	0.9–1.6	0.80	-	-	-
**Ever lived with a miner or former miner**	176/732	24.0	21.0–27.3	1.0	0.7–1.4	0.16	-	-	-
**Relationship with ex-miner or miner (ref: Friends/neighbor)**									
Spouse	41/151	27.2	20.4–35.1	0,78	0.5–1.4	0.38	-	-	-
Relatives	28/124	22.6	15.8–31.1			REF			
**No condom use at last sexual intercourse with regular partner, among male participants**	11/91	12.1	6.5–21.0	1.5	0.3–1.1	0.12	-	-	-
**No condom use at last sexual intercourse with regular partner, among female participants**	17/54	21.5	17.6–26.1	1.1	0.4–1.3	0.71	-	-	-
**Age of first sexual intercourse (Ref: >18)**									
<15	44/175	25,1	19.0–32.4	0.34	0.24–0.46	0.07	-	-	-
15–18	162/679	23.9	20.7–27.3	0.93	0.63–1.38	0.72	-	-	-

### Tuberculosis

From the total of 1012 participants, 1006 (99.4%) had one sputum sample collected, and it was submitted for a GeneXpert MTB/RIF test, 3 (0.3%)were MTB positive and sensitive to rifampicin. Of these 3, 2 were also co-infected with HIV. Overall, 7.5% participants reported having had TB in the past during their life, and 5 participants reported having tested positive for TB in the last 12 months and 2 were on treatment.

A very high proportion (95.0%) of participants were aware of TB, however, more than three-quarters (77.7%) of the respondents said they did not feel well informed about TB. Only 2.8% of respondents stated that TB bacteria can be prevented from spreading by covering the mouth and nose when coughing or sneezing (Table 5).

**Table 5:** TB history and knowledge in miners community of origin, Gaza Province, Southern of Mozambique, 2017.

Characteristic	n/N	%	(CI 95%)
**Ever heard about TB?**			
Yes	959/1012	95.0	93.4–96.2
**Fell well informed about TB**			
Yes	214/959	22.3	19.7–25.1
**First time heard about TB**			
Family, friends, neighbors and colleagues	541/959	56.4	53.2–56.6
Health workers	264/959	27.5	24.7–30.5
TV	29/959	3.0	2.1–4.4
Radio	16/959	1.7	1.0–2.8
Others	109/959	11.4	9.5–13.6
**Forms of TB prevention listed by respondents**			
Wash hands after touching items in public places	519 /959	51.4	48.3–54.6
Avoiding sharing dishes	459/959	45.5	42.4–48.6
Avoiding shaking hands	127/959	12.6	10.6–14.8
Covering the mouth and nose when cough or sneeze	28/959	2.8	1.9–4.0
**Ever had TB?**			
Yes	71/959	7.5	5.9–9.4
**TB tested in the last 12 months**			
Yes	30/959	2.9	1.8–9.7
**TB test results in the last 12 months**			
Positive	5/30	16.7	6.3–35.5
**TB treatment**			
Yes. Did it	3/5	40.0	7.3–83.0
Still doing the treatment	3/5	40.0	7.3–83.0

## Discussion

The results of this survey confirm that migrant miner’s communities of origin in southern Mozambique have a high HIV prevalence (24.2%). This is in agreement with the results of the Mozambique AIDS Indicator Survey, which points to a 24.4% HIV prevalence for the Gaza Province in the adult population of 15–49 years old, and ranked Gaza with the highest Prevalence for HIV [17]. This is also consistent with the first IBBS in miners conducted in the country [6].

The current study found a higher HIV prevalence among participants from the rural Muzingane community, compared to the urban community of Patrice Lumumba. This is in contrast with the results from the most recent national AIDS Indicator Survey which found that more infections occur in urban areas (16.8%) than rural areas (11.0%). However, the reasons for contrast could not be determined. In this study, factors for high prevalence in the rural area could be attributed to inadequate health infrastructure and education system in the rural community. Further research is needed to further investigate risk factors.

We observed that the HIV prevalence tend to increase with age and decline with increasing levels of education of participants. This is consistent with other study which showed HIV prevalence significantly increase in older age group [18]. Similar findings were also reported in the Mozambique AIDS Indicator Survey where prevalence was high among age group 35–39, and also higher among female with primary level of education (16.1%), and less in female without education (13.8%) [12]

The high proportion of persons unaware of their HIV positive status represents a substantial number of People living with HIV (PLHIV) who are not seeking care and treatment for their own health, and who are a potential source of new HIV infections [19].

Overall, it was observed that participants did not consistently use male condoms during last sexual intercourse with regular partner. It was striking that 28% of HIV-infected participants reported STIs, and 46% of males had reported two sexual partners in last 12 months before the survey. These findings all represent a serious behavior and biological risk factors, respectively to HIV acquisition and highlight the need to continually promote prevention interventions to limit HIV transmission in the Muzingane and Patrice Lumumba communities.

In this study, a prevalence of 0.3% TB was found. Although the prevalence of microbiologically confirmed TB was low, 7.5% reported having had TB at some point in their life. The number of active cases found is consisted with the figures found in previous studies conducted in similar areas and in other high TB burden areas in African settings [20–22]. This may not be the most cost effective way to find active TB cases, however 3 positive cases were diagnosed and linked to care which is significant since those were infectious cases [23]. In high burden settings, with limited resources, it is best to concentrate on household contacts and implement this outreach activity routinely to improve active case-finding.

Although many participants indicated that they have a high knowledge on modes of HIV and TB transmission, it was found that this included several misconceptions. Those gaps in knowledge highlight the need for increased interventions, such as promotional messages providing information on preventive and control practices. Although TB is a disease known to most participants, knowledge about prevention methods was low, which may have a negative impact on patients’ attitude towards health-seeking behavior and preventive methods [24].

Only 6.7% reported having heard of TB prevention programs on the radio, and for HIV the proportion was 26.2% for female and 51.4% for male participants. According to the 2011 Mozambique DHS report, of the three main mass media in Mozambique radio was the most cited source of information. As such, it is paramount not to overlook the critical role played by the radio on delivering HIV and TB related key intervention messages [25].

### Study limitations

Although this study contributes to the existing literature on HIV and TB prevalence on miner’s communities of origin in Mozambique, we acknowledge several limitations were encountered which should be considered when interpreting the findings. One limitation was the cross-sectional nature of the survey data, where it is not possible to establish temporality or causality. Given the survey procedures and the limitation of the study setting to mining communities in Gaza Province, the results cannot be extrapolated to other miners communities of origin due to regional differences, demographic and socio-economic conditions. There was no previous listing of individuals available for the interviews in the households, associated with the fact that the interviews took place between 8-12am, this may have contributed to why more females participated than men, since their male partners are working in a different location, and also the fact that many of the men are working outside the country. It is after all a community where men migrate frequently for work. The questionnaire used in this study used a skip pattern so that respondents did not respond to questions irrelevant to them, and this limited the interpretation of some results. Despite the high rates of completion of the forms, face-to-face interviews may have led to interviewer bias in the process of data collection and encouraged respondents to give socially desired responses, related to sexual behavior or other risk factors. Our questionnaire did not include questions on circumcision status, which can be critical in monitoring population level of Medical Male Circumcision (MMC) and its impact on HIV and other STI. Another limitation is that ART treatment status data was not collected from the participants in this study, so the survey was not able not analyze the proportion of PLHIV on treatment. Another potential limitation is related with TB diagnosis. The International Standards for Tuberculosis Care categorically states that all suspected of having pulmonary tuberculosis should have at least two, and preferably three sputum specimens preferably collected on successive days for microscopic examination to detect a large number of infectious cases in the community. On this study, only one sample was collected per patient [26,27]. In addition, sputum sample quality was not assessed and there may have been many samples which contained more salivary cells rather than actual sputum which contains pus cells from the source of inflammation.

## Conclusions

Gaza Province has the highest HIV prevalence rate in the country, and it is one of the provinces with high TB case notifications in the country. Given the high disease burden and low educational status, it is important to target the area to improve levels of awareness and promote consistent use of condom and health services that include screening and appropriate treatment for TB before miners are employed in the mines and when they are back from the mines on leave as well as systems that can trace and screen family members to prevent transmission and improve quality of care for this high-risk population. Also, it is advisable to employ strategies and interventions that encourage routine HIV testing, especially in healthcare settings, increase the availability of HIV testing services in non-medical settings, and improve knowledge on HIV and TB prevention, contributing to increasing the proportion of individuals who are aware of their HIV status, thus helping to control the epidemic.
